# Leucorrhea of Children

**Published:** 1858-01

**Authors:** 


### ARTICLE IV.

[TRANSLATED BY DR. BYFORD.]

### HOSPITAL FOR CHILDREN IN PARIS.—MAD. RICHARD. LEUCORRHEA OF CHILDREN.

You have seen, Gentlemen, how common an affection of little children is leucorrhea; we seldom have a clinic without noticing it. It is necessary to watch these little patients to have a just idea of the extreme abundance of this discharge. Nothing is more rebellious, and the worthy superintendent of Saint-Therese assures us that she has never seen a case cured. We had two of these little patients in this institution affected with this malady. In spite of their prolonged stay in the hospital and varied modes of treatment by M. Guersant and myself, they were in the same condition as when they first were admitted. In order better to ascertain the source of the discharge more than with a view to treatment, for I had despaired of success, three other little girls with this affection were admitted into the hospital. This made five. You find them in beds Nos. 5, 21, 23, 29 and 34, in the ward of Saint-Therese. M. Trousseau considers leucorrhea of children to consist generally of simple eczema of the vulva. We have satisfied ourselves that if there really is intertrigo of the vulva, it is simply produced by the discharge from the vagina, an effect rather than a cause. Does the mucous membrane of the uterus participate in the disease? What is the state of the weak? We have not arrived at a decision in these two respects.

However this may be, our five patients have been promptly cured, to the great astonishment of our pious nurse-mother of the ward. This success was effected by injections of colocynth. Dr. Claude, now of Paris, formerly of Verdun, is the author of an unpublished work on the effect of purgatives in general, and colocynth injections in particular. It is replete with originality, and is marked by real practical talent. As he cited me to a rebellious case of leucorrhea in a child cured by this means, I concluded to give it a trial in these cases, and, as you see, with surprising results. The treatment was instituted in the following manner, on a pod of colocynth of the ordinary size: two glasses of warm water was affused, and they were macerated for twenty-four hours. One-third of this liquid, after the colocynth was well expressed into it, is a dose for a child eight years old. Prior to using it, the rectum should be evacuated thoroughly by a large injection of tepid water. The time the colocynth injection will be retained is very variable, from fifteen minutes to an hour. During the day of its administration, the child will have from seven to thirty stools, the last of which are sanguinolent; the next day, from four to ten. Gum water is given to drink in any quantity the patient may wish. If food is desired, it should be very light, and given sparingly. The twelfth or thirteenth day the patient has entirely recovered from its effects and the appetite is good. On the sixteenth or seventeenth day, we may administer it again. Three of our patients have taken the colocynth injection three times, the other two four times. With all of them the discharge had very much diminished after the first injection; in three, complete suppression had taken place after the second. All five were retained over a fortnight after the cure had been entirely effected. We have seen three of them since, and have the address of all of them. The cure was complete and permanent.—*Gazette des Hopitaux*.

